# Supplementary figures and images for: Montreal Cognitive Assessment (MoCA) performance in Huntington’s disease patients correlates with cortical and caudate atrophy
Source: PeerJ. 2022 Apr 4;10:e12917. doi: 10.7717/peerj.12917 (PMC8988933; doi:10.7717/peerj.12917)

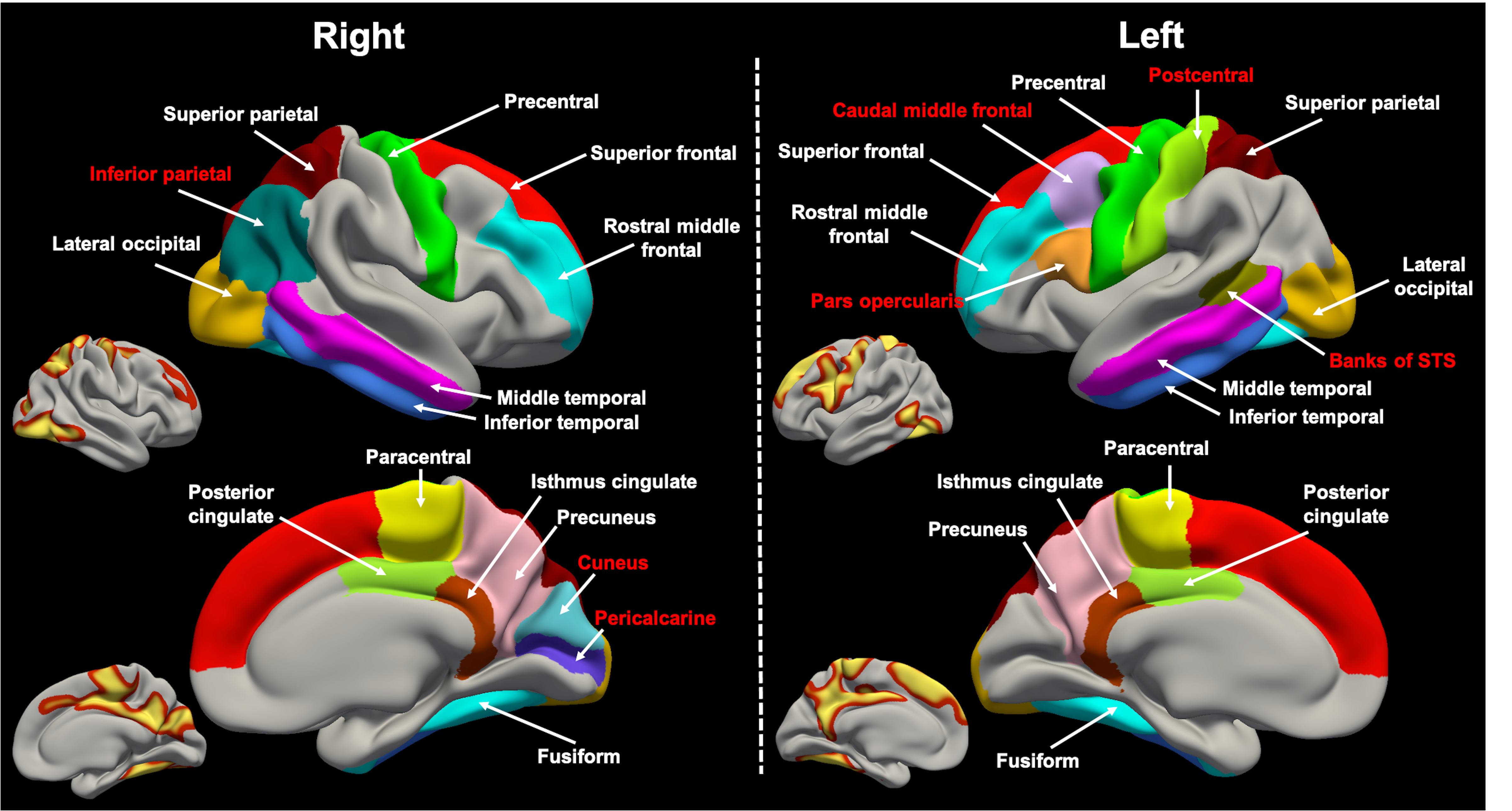

Supplement: Supplemental Information 1 — Cortical ROIs for each hemisphere are displayed with different colors to distinguish the areas covered by significant clusters. All ROIs are projected onto an average cortical surface and identified by cortical annotations. The anatomical regions specific for each hemisphere are displayed in red. Original significant cluster images are shown aside as appropriate. STS, superior temporal sulcus. [file peerj-10-12917-s001.png]
