# Supplementary material for: Montreal Cognitive Assessment (MoCA) performance in Huntington’s disease patients correlates with cortical and caudate atrophy
Source: PeerJ. 2022 Apr 4;10:e12917. doi: 10.7717/peerj.12917 (PMC8988933; doi:10.7717/peerj.12917)
Supplement: Supplemental Information 2 [file peerj-10-12917-s002.docx]

| **Right hemisphere** | | | | | |
| --- | --- | --- | --- | --- | --- |
| **Anatomical region** | **Peak max value** | **Size (mm^2^)** | **Talairach coordinates** | | |
|  |  |  | **X** | **Y** | **Z** |
| Precuneus | 4.230 | 6963.60 | 7.7 | -53.6 | 26.1 |
| Lateral occipital | 5.698 | 4768.01 | 44.5 | -71.8 | -8.2 |
| Inferior parietal | 4.211 | 4573.84 | 31.8 | -57.0 | 44.9 |
| Rostral middle frontal | 3.307 | 2169.93 | 33.1 | 39.9 | 19.2 |
