# Supplementary material for: Montreal Cognitive Assessment (MoCA) performance in Huntington’s disease patients correlates with cortical and caudate atrophy
Source: PeerJ. 2022 Apr 4;10:e12917. doi: 10.7717/peerj.12917 (PMC8988933; doi:10.7717/peerj.12917)
Supplement: Supplemental Information 3 [file peerj-10-12917-s003.docx]

| **Left hemisphere** | | | | | |
| --- | --- | --- | --- | --- | --- |
| **Anatomical region** | **Peak max**  **value** | **Size (mm^2^)** | **Talairach coordinates** | | |
|  |  |  | **X** | **Y** | **Z** |
| Superior frontal | 5.931 | 9325.11 | -19.3 | 35.4 | 42.8 |
| Precuneus | 5.332 | 5362.73 | -4.8 | -58.2 | 24.4 |
| Fusiform | 5.258 | 4468.02 | -41.1 | -42.4 | -12.4 |
