# Supplementary material for: Montreal Cognitive Assessment (MoCA) performance in Huntington’s disease patients correlates with cortical and caudate atrophy
Source: PeerJ. 2022 Apr 4;10:e12917. doi: 10.7717/peerj.12917 (PMC8988933; doi:10.7717/peerj.12917)
Supplement: Supplemental Information 4 [file peerj-10-12917-s004.docx]

| **MoCA test correlations** | ***r* coefficient** | ***p*-uncorrected** | ***p-*value**  **(Bonferroni correction)** |
| --- | --- | --- | --- |
| Caudate R | 0.142 | .550 | 1 ^NS^ |
| Caudate L | 0.214 | .364 | 1 ^NS^ |
| Putamen R | -0.044 | .852 | 1 ^NS^ |
| Putamen L | 0.222 | .345 | 1 ^NS^ |
| Thalamus R | 0.200 | .395 | 1 ^NS^ |
| Thalamus L | -0.275 | .239 | 1 ^NS^ |
| Globus Pallidus R | 0.0003 | .998 | 1 ^NS^ |
| Globus Pallidus L | 0.407 | .074 | 1 ^NS^ |
| Hippocampus R | -0.057 | .809 | 1 ^NS^ |
| Hippocampus L | 0.211 | .370 | 1 ^NS^ |
| Amygdala R | 0.074 | .755 | 1 ^NS^ |
| Amygdala L | 0.461 | .040 | 1 ^NS^ |
| Accumbens R | 0.248 | .291 | 1 ^NS^ |
| Accumbens L | 0.142 | .550 | 1 ^NS^ |
