# Supplementary material for: Montreal Cognitive Assessment (MoCA) performance in Huntington’s disease patients correlates with cortical and caudate atrophy
Source: PeerJ. 2022 Apr 4;10:e12917. doi: 10.7717/peerj.12917 (PMC8988933; doi:10.7717/peerj.12917)
Supplement: Supplemental Information 5 — N/T, no treatment; N/A, no available [file peerj-10-12917-s005.docx]

| **HD patient ID** | **Medication** | **Dosage** | **Usage** |
| --- | --- | --- | --- |
| HD001 | N/T | -- | -- |
| HD002 | N/T | -- | -- |
| HD003 | N/T | -- | -- |
| HD004 | N/T | -- | -- |
| HD005 | N/T | -- | -- |
| HD006 | Olanzapine | 5 mg once daily (1 tab) | Antidepressant |
|  | Venlafaxine | 75 mg three times daily 8 h interval (3 tabs) | Antidepressant |
|  | Metformin | 500 mg three times daily 8 h interval (3 tabs) | Antidiabetics – antihyperglycemic |
| HD007 | Calcium and vitamin D | N/A – 1 tab daily | Supplement |
|  | Multivitamins | N/A – 1 tab daily | Supplement |
| HD008 | Citalopram | 20 mg once daily (1 tab) | Antidepressant |
|  | Coenzyme Q10 | N/A – 1 tab daily | Supplement |
| HD009 | Amantadine | 50 mg three times daily 8 h interval (3 tabs) | Antidyskinetic |
| HD010 | N/T | -- | -- |
| HD011 | N/T | -- | -- |
| HD012 | N/T | -- | -- |
| HD013 | N/T | -- | -- |
| HD014 | Tetrabenazine | 25 mg once daily (1 tab) | Antihyperkinetic movement – Chorea |
|  | Coenzyme Q10 | N/A – 1 tab daily | Supplement |
| HD015 | Omega-3 Fatty Acids | N/A – 1 tab daily | Supplement |
|  | Coenzyme Q10 | N/A – 1 tab daily | Supplement |
| HD016 | -- | -- | -- |
| HD017 | Citalopram | 20 mg once daily (1 tab) | Antidepressant |
|  | Risperidone | 0.5 mg once daily (1 tab) | Antipsychotic |
| HD018 | Haloperidol | 0.3 mg three times daily 8 h interval (1 mL in total) | Antipsychotic |
|  | Paroxetine | 40 mg once daily (1 tab) | Antidepressant |
| HD019 | N/T | -- | -- |
| HD020 | N/T | -- | -- |
| HD021 | N/T | -- | -- |
| HD022 | N/T | -- | -- |
